# Genomics of Compensatory Adaptation in Experimental Populations of *Aspergillus nidulans*

**DOI:** 10.1534/g3.116.036152

**Published:** 2016-11-29

**Authors:** Jeremy R. Dettman, Nicolas Rodrigue, Sijmen E. Schoustra, Rees Kassen

**Affiliations:** * Department of Biology, University of Ottawa, Ontario K1N 6N5, Canada; † Centre for Advanced Research in Environmental Genomics, University of Ottawa, Ontario K1N 6N5, Canada; ‡ Department of Biology, Carleton University, Ottawa, Ontario K1S 5B6, Canada; § Laboratory of Genetics, Wageningen University, NL-6708 PB, The Netherlands

**Keywords:** fungicide resistance, adaptive walks, trade-offs, fludioxonil

## Abstract

Knowledge of the number and nature of genetic changes responsible for adaptation is essential for understanding and predicting evolutionary trajectories. Here, we study the genomic basis of compensatory adaptation to the fitness cost of fungicide resistance in experimentally evolved strains of the filamentous fungus *Aspergillus nidulans*. The original selection experiment tracked the fitness recovery of lines founded by an ancestral strain that was resistant to fludioxonil, but paid a fitness cost in the absence of the fungicide. We obtained whole-genome sequence data for the ancestral *A. nidulans* strain and eight experimentally evolved strains. We find that fludioxonil resistance in the ancestor was likely conferred by a mutation in histidine kinase *nikA*, part of the two-component signal transduction system of the high-osmolarity glycerol (HOG) stress response pathway. To compensate for the pleiotropic negative effects of the resistance mutation, the subsequent fitness gains observed in the evolved lines were likely caused by secondary modification of HOG pathway activity. Candidate genes for the compensatory fitness increases were significantly overrepresented by stress response functions, and some were specifically associated with the HOG pathway itself. Parallel evolution at the gene level was rare among evolved lines. There was a positive relationship between the predicted number of adaptive steps, estimated from fitness data, and the number of genomic mutations, determined by whole-genome sequencing. However, the number of genomic mutations was, on average, 8.45 times greater than the number of adaptive steps inferred from fitness data. This research expands our understanding of the genetic basis of adaptation in multicellular eukaryotes and lays out a framework for future work on the genomics of compensatory adaptation in *A. nidulans*.

A long-standing question in evolutionary biology concerns the number of genetic changes responsible for adaptation. A common view was that adaptation was underlain by many genes, each having small individual effects on fitness (Fisher 1930; Orr 1998). This interpretation initially gained wide acceptance, partly because there was little evidence with which it could be confronted (Orr 2005). A powerful approach for the study of the genetic basis of adaptation is through microbial selection experiments, especially when coupled with cost-effective whole-genome sequencing (Elena and Lenski 2003; Long *et al.* 2015). Not only can adaptive mutations be tracked in real time as they arise, but the number and nature of genetic changes underlying adaptive evolution can be determined directly.

A compelling result to emerge from this field of work is that much of the fitness gain associated with an adaptive walk (sequential substitution of beneficial mutations by selection) is attributable to a small number of genetic changes (*e.g.*, Wichman *et al.* 1999; Riehle *et al.* 2001; Conrad *et al.* 2009; Schoustra *et al.* 2009; Anderson *et al.* 2010; Kvitek and Sherlock 2011). A recent review, for example, showed that the number of mutations fixed in microbial selection experiments that have reached a fitness plateau is typically between one and four (Kassen 2014). This result is consistent with the distribution of fitness effects among beneficial mutations being highly variable, such that in large populations, initial increases in fitness are associated with mutations that have large beneficial effects. Nonetheless, this result likely represents an underestimate because many analyses infer the number of fixation events from fitness trajectories or genetic crosses rather than whole-genome sequencing. Moreover, most microbial experiments are conducted using very large population sizes that violate one of the key assumptions of models of adaptation: that beneficial mutations are rare events that, when they occur, sweep nearly instantaneously to fixation. Consequently, the number of genetic changes underlying adaptive walks may be substantially higher than previous estimates have inferred.

To gain a deeper understanding of the genetic basis of adaptation, we sequenced the genomes of experimentally evolved lines of the filamentous fungus *Aspergillus nidulans*. These lines were generated during a recently reported selection experiment (Schoustra *et al.* 2009) in which 118 selection lines were founded by a common ancestor (strain WG615) that carried a mutation that conferred resistance to the fungicide fludioxonil. As is often the case, a pleiotropic effect of the resistance mutation was a strong fitness cost in the absence of the fungicide (46% reduced growth rate; Schoustra *et al.* 2006). To allow for compensatory evolution, the populations were propagated in a fungicide-free environment for 800 generations with two different effective population sizes. The recovery of fitness was tracked during the course of the selection experiment, and all lineages reached a fitness plateau by the end, suggesting that the bulk of fitness recovery had been accomplished. From data on the fitness trajectories, a novel maximum likelihood framework and genetic crosses were used to infer the number of beneficial, compensatory mutations. The main finding was that adaptive walks tended to be rather short, with an overall mean of only 2.20 steps (Schoustra *et al.* 2009).

The exact molecular mode of action of phenylpyrrole fungicides such as fludioxonil has yet to be elucidated, but they are known to act through the disruption of the HOG response pathway (Furukawa *et al.* 2007; Hagiwara *et al.* 2007, 2009, 2013). This pathway involves a two-component system and a mitogen-activated protein kinase (MAPK) signaling cascade, in which a histidine kinase serves as a sensor for multiple stressors and transmits the signal downstream via a response regulator to activate the MAPK. Previous work with *Aspergillus* has shown this stress response pathway to be activated by exposure to fludioxonil (Furukawa *et al.* 2007; Hagiwara *et al.* 2009) and that knocking out components of this pathway results in high levels of fludioxonil resistance (Hagiwara *et al.* 2007, 2013). Genes involved in the HOG MAPK pathway are therefore prime candidates for fludioxonil resistance determinants.

Here, we sequence the 30.5 Mb genomes of the ancestral *A. nidulans* strain and eight experimentally evolved strains (Schoustra *et al.* 2009) to investigate the evolutionary genomics of compensatory adaptation to the fitness cost of fungicide resistance. First, since the genetic basis of the fungicide resistance is not known, we identify candidate mutations for resistance in the ancestral strain. We then identify mutations within the experimentally evolved strains and analyze the characteristics of the mutated genes to determine how they may be related to compensatory, adaptive evolution. Finally, we use these data to determine the relationship between the number of genomic mutations and the predicted number of adaptive steps estimated from fitness data, and we also test hypotheses about how effective population size impacts the number and nature of mutations fixed during adaptation.

## Materials and Methods

### Strains

The ancestral *A. nidulans* strain is fludioxonil-resistant WG615 (*fldA1*, *wA3*, *pyroA4*, and *veA1*). When growing in the absence of fungicide, resistance confers a cost of ∼46% relative to the sensitive strain from which WG615 was derived (Schoustra *et al.* 2006). All evolved strains were derived from WG615 through 800 generations of experimental evolution and adaptation to a permissive growth environment [complete medium (CM) without fungicide; Schoustra *et al.* 2009]. The wild-type sensitive strain was not evolved because, in previous experiments (Schoustra *et al.* 2005, 2006), it showed little evidence of adaptation to the permissive growth environment, suggesting that the sensitive strain already resides close to a fitness optimum. Every 80 generations, experimental lines were put through a bottleneck of either ∼500 or 50,000 spores, corresponding to small (S) and large (L) effective population size treatments, respectively. For whole-genome sequencing, we chose four strains from each of the small and large population size treatments. Representative strains were chosen to cover the full range of observed fitness values and predicted step numbers (Supplemental Material, Table S1). The eight evolved strains characterized here (Table S1) were all put through a single-conidium transfer to ensure that a single haploid genotype was being sequenced. From the results of Schoustra *et al.* (2009), the number of predicted adaptive steps per strain ranged from one to three.

### DNA extraction and sequencing

Strains were grown on CM agar plates and genomic DNA was extracted using the QIAGEN DNeasy Blood & Tissue Kit. Sequence data were generated using the Illumina Hi-Seq platform with paired-end reads (BC Cancer Agency). Reads generated for the evolved and ancestral strains were 75 and 100 bp in length, respectively.

### Reference-based mapping

The *A. nidulans* reference genome was from strain FGSC A4 and is publicly available from NCBI (PRJNA13961). The assembly is divided into scaffolds (NT_106999 to NT_1070015) corresponding roughly to the 16 arms of the eight *A. nidulans* chromosomes. A modified version of the bioinformatics pipeline described in Dettman *et al.* (2012) was used for sequence data analyses. In brief, reads were trimmed using Popoolation (ver. 1.1, Kofler *et al.* (2011)) with a phred quality threshold of 20 and a minimum retention length of 75% of original read length. Trimmed reads were mapped to the FGSC A4 reference genome using Novoalign (ver. 2.07).

### Coverage

Coverage along scaffolds was calculated in 1 kb intervals and regions of high or low coverage were identified by bins with >1.8 × or <0.20 × the average coverage of the entire genome of that sample. Regions were confirmed by manual inspection of aligned reads and by results from other insertion/deletion (indel) calling programs (Pindel, ver. 0.2.4, Ye *et al.* 2009; Breseq, ver. 0.16, Barrick *et al.* 2014). Areas of high or missing coverage were reported if they were >500 bp in length, and regions that were within 200 bp of each other were collapsed into one.

### Variant calling

Single nucleotide polymorphisms (SNPs) and indels relative to the reference were called using Samtools (ver. 0.1.19, Li *et al.* 2009; minimum coverage = 3 ×), VarScan (ver. 2.3.5, Koboldt *et al.* 2012; minimum coverage = 3 ×, *P* > 0.95), and Pindel (ver. 0.2.4). Results from the different methods were compared for cross-verification of mutation calls. A variant shared by WG615 and all evolved lines indicates that that variant was present in the ancestral WG615 strain at the start of the experiment. Variants that arose during the selection experiment were those that were absent in WG615 but present in an evolved line. For variant calling, we filtered out the regions of low quality assembly in the reference genome, and correspondingly, regions of low mapping quality in the query genomes. Therefore, we may have underestimated the true number of sequence differences between the strains. Sequence data and positional alignments were inspected manually to confirm the called mutations in evolved lines. Significance of Pearson’s correlations were determined using the Student’s *t*-distribution.

### Annotation and functional analyses

Genomic variation was annotated using SnpEff (ver. 3.3, Cingolani *et al.* 2012, minimum coverage = 3 ×, minimum base quality = 20) to determine the types of mutation and what genes were affected. Amino acids were classified as hydrophilic uncharged (S, N, T, and Q), aliphatic uncharged (A, G, V, L, and I), nonpolar uncharged (C, M, and P), acidic (D and E), basic (K, R, and H), and aromatic (F, Y, and W). Amino acid changes within and between these classes were considered conservative and radical, respectively. Mutations that caused codon indels, premature stop codons, or frame-shifts were also considered radical. Intergenic mutations were investigated further if they occurred in a 5′-untranslated region or within 100 bp upstream of the transcriptional start site of a gene. Gene functions were inferred, in part, from automated Gene Ontology (GO) information. To summarize the functional information using broad, high-level GO terms, we used the GO Slim sets provided by the *Aspergillus* Genome Database (www.aspergillusgenome.org). For enrichment analyses, we aimed to identify the main functional classes, so the results were filtered to retain only the GO Slim terms that were represented by at least 5% of the queried genes. A GO Slim term was considered overrepresented if the observed gene number was ≥ 1.2 times the expected gene number. Statistical significance of overrepresentation was calculated using one-tailed Chi-squared tests and the Benjamini–Hochberg false discovery rate (set to 0.15) correction for multiple comparisons. Given that “Cellular Component,” “Biological Process,” and “Molecular Function” GO Slim annotations were missing for 57, 56, and 54% of genes, respectively, we performed more comprehensive manual annotation of all affected genes using information from other sources, such as similarity and literature searches.

### Data availability

Sequence data are available from the NCBI Short Read Archive under BioProject PRJNA356622.

## Results and Discussion

### Sequence data

Sequence libraries contained an average of 35.8 million reads per sample, providing an expected 89.4 × coverage of the 30.5 Mb *A. nidulans* genome (Galagan *et al.* 2005). To reduce the number of false positive variant calls in downstream analyses, sequence reads were trimmed based on quality criteria. Trimming reduced the average read number and read length by only 4.3 and 1.1%, respectively, indicating that the overall sequence quality was relatively high (Table S1). All subsequent analyses were based on trimmed reads.

### Coverage distribution

An average of 93.7% of reads from the ancestor WG615 and the eight evolved lines were successfully mapped to the FGSC A4 reference genome, resulting in an average coverage of 83.1 × (Table S1). Comparison of fold-coverage among and along scaffolds revealed that WG615 and all descendent, evolved lines shared numerous regions of missing coverage relative to ancestral strain FGSC A4 (Table S2). These absent regions could be up to 32.5 kb in length but still only comprised 1.7% of the reference genome (504.4 kb total). Also, some genome regions of poor alignment and/or low mapping quality were identified (Table S3). It is not clear if these areas represent genomic regions of poor reference assembly, repetitive regions, or regions of genome plasticity. Regardless, these low quality regions sum to only 82.6 kb, or 0.27% of the reference genome, and therefore have a negligible effect on our mutation detection ability. When filtering for changes that arose during the selection experiment, no evidence for large scale indels or aneuploidies were found.

### Variant calls for ancestral WG615 strain

Variant calling revealed that the ancestral WG615 strain differed from FGSC A4 reference by 740 SNPs and 456 small indels, confirming that these two strains have moderate sequence divergence (0.004%). WG615 is described as having mutations in the *wA* (AN8209), *pyroA* (AN7725), and *veA* (AN1052) genes, so we examined these genes for quality control purposes. Mutations were confirmed for *wA* and *pyroA*, and WG615 and FGSC A4 shared the same *veA* mutant allele, as predicted.

### Candidate genes for fungicide resistance in ancestral WG615 strain

To identify putative targets of fludioxonil resistance, we took a two-step strategy. We first filtered sequence differences between the fludioxonil-resistant WG615 strain and the fludioxonil-sensitive FGSC A4 strain to retain only those that caused protein sequence changes, under the assumption that these were the most likely to cause changes in gene function and phenotype. Filtering narrowed the field to 390 mutations, of which 237 had GO annotation information available. We screened for genes involved in the HOG MAPK pathway because they are prime candidates for fludioxonil resistance determinants. Just two of these candidate genes (AN2262 and AN4479) had described osmoregulation functions, and only the latter gene, AN4479, is known to respond to fungicides. Notably, AN4479 encodes *nikA*, the histidine kinase sensor of the two-component system of the HOG MAPK pathway.

Second, we reviewed our previous genetic analyses (Schoustra *et al.* 2006) and found that the resistance mutation was located on the left arm of *A. nidulan*s linkage group III, between the centromere and *cys2* (AN4769; also known as sC or *sC*0). Examination of our genomic data revealed that only 12 genes in this genomic region had predicted protein differences between the sensitive reference and resistant WG615 ancestor. Inspection of annotation information for these 12 candidate genes (Table S4) revealed that only one had known functions related to osmoregulation or fungicide resistance: that one gene was *nikA*.

Three lines of evidence support that *nikA* is the target of fludioxonil resistance in WG615. First, *nikA* is essential for fludioxonil-based growth inhibition (Hagiwara *et al.* 2007, 2013; Vargas-Perez *et al.* 2007) and is responsible for transmitting the phosphorelay signal down the HOG MAPK pathway (Hagiwara *et al.* 2009). Second, *nikA* mutants with increased resistance to fludioxonil are characterized by strong vegetative growth defects in the absence of the fungicide (Hagiwara *et al.* 2007; Vargas-Perez *et al.* 2007; De Souza *et al.* 2013), a fitness trade-off clearly observed in our fludioxonil-resistant strain (Schoustra *et al.* 2005, 2009). Third, the WG615 *nikA* mutation is a nonsynonymous substitution causing a radical amino acid change (K→N) at residue 854, which is within the highly conserved, signal transduction histidine kinase domain. Although formal genetic analyses are needed for full confirmation, the evidence points to the *nikA* mutation as the determinant of fludioxonil resistance.

### Derived mutations in compensatory evolution lines

**Figure 1:**
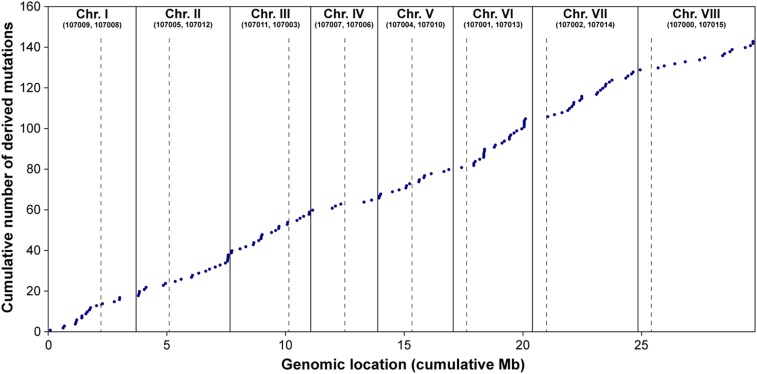
Cumulative number of derived mutations and their locations within the *A. nidulans* genome. Data for all eight evolved lines were combined. Chromosome (Chr.) ends are indicated by solid lines and centromere positions are indicated by dashed lines. Chromosome numbers and associated genome sequence scaffolds (FGSC A4) are listed at the top.

We identified 144 SNPs and indels that differed between the ancestor and the compensatory evolution lines after 800 generations of selection (Table 1, Table S5, and Table S6). An average of 16.75 SNPs and 1.25 small indels were detected in the ∼30.5 Mb genome of each evolved line. Derived mutations were relatively evenly distributed throughout the genome (Figure 1), with only a few locations containing multiple mutations clustered together. Only one derived structural variant was called: a ∼103 kb inversion was predicted in strain 25S (Table S6B). Substitution rates per chromosome did not differ drastically and ranged from 3.32 to 11.66 × 10^−10^ per nucleotide per generation (Table S7). The overall substitution rate was 7.58 × 10^−10^ per nucleotide per generation, similar to rates reported for other fungi such as yeast (Lynch *et al.* 2008; Nishant *et al.* 2010). Approximately 50% of the *A. nidulans* genome is coding, yet over 57% of the observed mutations (83/144) were located within coding regions (Table 1). This difference between observed and expected proportions in coding regions is not statistically significant (Chi-square, *P* < 0.194), but the overrepresentation of coding mutations is consistent with positive selection in our experiment.

**Table 1:** Types and numbers of derived mutations in evolved lines

Mutation Type	Small (S) Population Size					Large (L) Population Size				
	9S	16S	25S	42S	S Total (%)	8L	37L	45L	59L	L Total (%)
Intergenic	14	6	5	6	31 (38.3)	3	4	10	9	26 (41.3)
Intron	1	1	0	1	3 (3.7)	0	1	0	0	1 (1.6)
Coding, synonymous	5	1	4	7	17 (21.0)	3	1	4	3	11 (17.5)
Coding, nonsynonymous	9	10	0	6	25 (30.9)	4	5	7	6	22 (34.9)
Coding, other*^a^*	2	0	2	1	5 (6.2)	0	1	1	1	3 (4.8)
All mutations	31	18	11	21	81	10	12	22	19	63

a Includes codon change with codon insertion, stop gained, and frame-shift mutations.

### Genes mutated during compensatory adaptation

The HOG MAPK pathway in *A. nidulans* senses and responds to multiple forms of potentially harmful stimuli, such as osmotic stress, oxidative stress, DNA damage, and fungicides (Furukawa *et al.* 2007; Miskei *et al.* 2009; Balazs *et al.* 2010; Jaimes-Arroyo *et al.* 2015). If the *nikA* resistance-conferring mutation disrupts the normal function of the HOG MAPK signaling pathway, there are likely to be pleiotropic effects on several other physiological processes and phenotypes (*e.g.*, Vargas-Perez *et al.* 2007; Duran *et al.* 2010). Our observation of a substantial fitness cost associated with resistance is consistent with this idea. Recent studies of compensatory adaptation in yeast (Szamecz *et al.* 2014; Filteau *et al.* 2015) found that derived genetic changes were often specific to the original functional defect, and that compensatory evolution targeted genes that were functionally related to the disrupted gene. Thus, the obvious prediction was that the compensatory fitness gains observed here in *A. nidulans* would be caused by secondary modification of the HOG MAPK pathway.

Genome-wide analyses in *A. nidulans* are hampered by a lack of complete functional annotation for genes, particularly when compared to bacterial genomes or more intensively studied fungi like yeast. For example, the most recent *A. nidulans* genome snapshot indicates that only ∼11% of ORFs are verified, while the rest remain uncharacterized. Given the high frequency of missing data, our power to detect statistically significant trends was quite low. However, such results are still useful for identifying potential functional pathways under selection and candidate genes to target in future research efforts.

**Figure 2:**
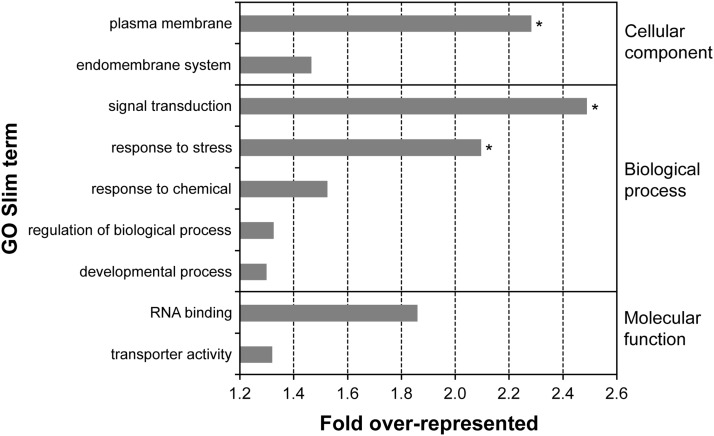
Summary of the Gene Ontology (GO) Slim terms that were overrepresented (>1.2-fold) in the list of genes that were mutated during compensatory adaptation. Terms that were significantly overrepresented after correction for multiple comparisons are marked by an asterisk (*).

For *A. nidulans*, the main source for functional information is GO data. We used GO information (Table S5), summarized using GO Slim Mapper (Table S8), to identify the main functional categories of genes affected by derived mutations. To determine if particular functional classes were more prevalent than expected by chance, the frequencies of genes belonging to specific GO Slim terms were compared between the mutated gene list and that for the rest of the genome. Despite the fact that less than half of the mutated genes (38/85) had GO information, we were still able to identify three GO Slim terms that were significantly overrepresented (after correction for multiple comparisons) in the mutated gene list (Figure 2). The relevance of these three important functional classes, and examples of specific genes, are discussed below. Interestingly, the enriched functional classes were consistent with the HOG MAPK pathway’s role as a stress response pathway with phosphorelay-based transduction of signals to downstream elements.

#### Response to stress:

Genes associated with “response to stress” (GO 6950) were overrepresented 2.1-fold among derived mutations (Chi-square, *P* < 0.003). We examined the affected genes and found some that were specifically associated with the HOG MAPK pathway itself (Table 2). The most obvious example was the *atfA* gene (AN2911), which encodes a transcription factor in the HOG MAPK pathway and regulates responses to a broad spectrum of environmental stressors (Hagiwara *et al.* 2009; Balazs *et al.* 2010; Lara-Rojas *et al.* 2011). The derived *atfA* mutation was a radical nonsynonymous change (Q→P) of a conserved amino acid within the functionally important basic-region leucine zipper (bZIP) domain. Another mutated gene with a direct role in the central stress response pathway is *mpkC*, which encodes a second MAPK that is activated by the same upstream MAPKK as *hogA* (= *sakA*) MAPK. Overexpression of *mpkC* has been shown to suppress the high-osmolarity sensitivity observed in *hogA*-deletion strains (Furukawa *et al.* 2005), indicating that these two homologous genes have overlapping roles in a shared pathway. No compensatory mutations were observed in *nikA* itself.

**Table 2:** Examples of mutated genes with functions relevant to stress response or membrane and cell wall

Gene	Mutation Type	Gene Product	Gene Function
Stress response			
AN2911	Nonsynonymous	atfA, basic-region leucine zipper transcription factor activated by HOG MAPK	Stress response (osmotic, fungicide, oxidative)
AN3101	Nonsynonymous	phkB, histidine kinase D5, stress-activated phosphotransfer protein	Phosphorelay sensor kinase activity, stress-activated (osmotic, oxidative, and fungicide)
AN3793	Nonsynonymous	ppzA, serine/threonine-protein phosphatase, catalytic subunit of protein phosphatase Z (PPZ)	Resistance to oxidative stress
AN4668	Synonymous	mpkC, MAPK, highly similar to HogA	Response to osmotic stress, development
AN2204	Nonsynonymous	Osmoadaptation protein with unknown function	Cellular response to osmotic stress
AN4789	Nonsynonymous	uvsI, DNA polymerase	Response to UV-damage of DNA, postreplication DNA repair
AN8562	Nonsynonymous	Ankyrin repeat protein	Stress response (camptothecin)
AN4206	Nonsynonymous	DnaJ domain protein	Protein folding and chaperone binding, stress response (camptothecin)
AN1217	Nonsynonymous	Putative LIM/homeobox transcription factor	Stress response (camptothecin)
AN6280	Nonsynonymous	Uncharacterized	Stress response (camptothecin)
AN5217	Codon change plus codon insertion	pilA, putative eisosome component	Protein localization, stress response (camptothecin)
AN6753	Synonymous	Putative NADH-dependent flavin oxidoreductase	Stress response (menadione)
AN1700	Synonymous	Putative 26S proteasome regulatory subunit Rpn2	Proteasome assembly, stress response (camptothecin)
Membrane and cell wall			
AN3307	Nonsynonymous	agsB, catalytic subunit of the major α-1,3 glucan synthase complex	Glucan synthesis, osmotic stress, and cell wall integrity
AN3504	Nonsynonymous	Putative α-1,4-glucosidase	Starch and disaccharide degradation
AN7396	Nonsynonymous	bglM, putative β-glucosidase	Cellulose and polysaccharide degradation
AN8765	Frame-shift	Activator of chitin synthase	Response to osmotic stress, regulation of chitin synthase activity and cell wall formation
AN4686	Synonymous	csnA, chitosanase	Endo-chitosanase activity, predicted glycosyl phosphatidylinositol (GPI)-anchor
AN3247	Frame-shift	ABC multidrug transporter	Predicted ATP binding, ATPase activity, coupled to transmembrane movement of substances
AN2287	Nonsynonymous	Putative transmembrane transporter	Predicted amino acid transport activity, GABA transport activity
AN0660	Nonsynonymous	furA, putative nucleobase and allantoin transporter	Induced by allantoin and by uric acid
AN2699	Nonsynonymous	Uncharacterized	Predicted transmembrane transport activity, integral component of membrane localization
AN3247	Synonymous	ABC multidrug transporter	Predicted ATP binding, ATPase activity, coupled to transmembrane movement of substances
AN5370	Synonymous	Putative MFS multidrug transporter	Predicted transmembrane transport
AN3113	Synonymous	ugtA, UDP-galactofuranose transporter, DMT family organic anion transporter, multidrug resistance efflux domain	Cell wall architecture, hyphal morphology, and drug sensitivity
AN10396	Synonymous	Putative farnesyl-diphosphate farnesyltransferase	Lipid biosynthetic process

See Table S6 for details on specific mutations. HOG, high-osmolarity glycerol; MAPK, mitogen-activated protein kinase; UV, ultraviolet; NADH, nicotinamide adenine dinucleotide hydride; ATP, adenosine triphosphate; GABA, γ-aminobutyric acid; MFS, major facilitator superfamily; UDP, uridune diphosphate; DMT, drug/metabolite transporter.

Several mutated genes (Table 2) are known to be expressed in *Aspergillus* at increased levels in response to osmotic stress or stress-inducing chemicals such as menadione and camptothecin (Malavazi *et al.* 2006). Camptothecin is a cytotoxic quinoline alkaloid that contains a pyrrole moiety, which is a defining characteristic of phenylpyrrole fungicides such as fludioxonil. This shared chemical structure may explain why both compounds appear to activate similar stress response pathways.

#### Signal transduction:

Genes associated with “signal transduction” (GO 7165) were overrepresented 2.5-fold among derived mutations (Chi-square, *P* < 0.029), many of which had direct roles in the phosphorylation-based transduction of stress response signals. For example, a radical nonsynonymous mutation (S→F) was observed in *ppzA*, the catalytic subunit of protein phosphatase Z. This serine/threonine phosphatase is known to modulate the response of fungi to oxidative and other stresses (Leiter *et al.* 2012). A radical nonsynonymous mutation (T→I) was also found in the histidine kinase *phkB*. Homologs of *phkB* are stress-activated sensor kinases of two-component systems, indicating a role in phosphorylation-based signal transduction, and a recent study with *Aspergillus* found that *phkB* expression increased greatly in response to fludioxonil treatment and osmotic shock in a manner dependent on the HOG pathway (Hagiwara *et al.* 2013).

#### Plasma membrane:

Genes associated with the “plasma membrane” (GO 5886) were overrepresented 2.3-fold among derived mutations (Chi-square, *P* < 0.026). Remodeling of the fungal membrane and cell wall components is a common response to a wide range of stressors because it helps maintain cell wall integrity and allow for survival and growth (Ene *et al.* 2015). We found derived mutations in a number of genes (Table 2) with functions in the synthesis and breakdown of fungal cell wall components, such as glucans (*agsB*, *bglM*, and AN3504) and chitin (*csnA* and AN8765; de Groot *et al.* 2009). For example, a nonsynonymous mutation was observed in the main catalytic domain of *agsB*, which encodes an α-1,3 glucan synthase with roles in osmotic stress tolerance and cell wall integrity (Futagami *et al.* 2011). The mutated gene list also contained several membrane-bound transporters with specificities for a range of compounds. Transporters are known to mediate resistance to fungicides and other toxic compounds in many fungi, including *Aspergillus* (Del Sorbo *et al.* 2000).

#### Intergenic mutations:

Intergenic mutations can alter 5′-untranslated regions (5′-UTRs) or promoter sequences, potentially resulting in changes in gene expression that affect fitness. We analyzed the 57 intergenic mutations (Table S6) and found five that were within 5′-UTRs, and eight that were within potential promoter regions (defined as < 100 bp from the transcription start site of a gene). None of the potentially affected genes had known roles in the HOG MAPK pathway, osmotic stress tolerance, or phenylpyrrole resistance.

### Parallel evolution

Parallel evolution is the repeated evolution of the same genotype or phenotype in independently evolving populations. At the level of fitness, all of the lines we studied evolved in parallel; that is, they all increased in fitness substantially when compared to their ancestor (Schoustra *et al.* 2009). Only one of the derived, mutated genes was hit in multiple evolved lines (uncharacterized gene AN2078, predicted RNA polymerase II transcription cofactor activity; strain 9S and 8L, Table S5), providing little evidence for parallel evolution at the gene level. There may be multiple evolutionary pathways to increased fitness in the permissive environment, yet empirical evidence suggests that compensatory evolution would target pathways that were functionally related to the disrupted pathway that caused the original fitness deficit (Szamecz *et al.* 2014; Filteau *et al.* 2015).

Current understanding of the HOG pathway [KEGG (Kyoto Encyclopedia of Genes and Genomes), Furukawa *et al.* 2005; Hagiwara *et al.* 2009] indicates that it involves 22 of the 10,667 protein-coding genes in *A. nidulans*. We observed derived mutations in only two of these genes (mpkC and atfA), but the probability of 2/87 derived mutations occurring randomly in this gene subset is extremely small (∼3.57 × 10^−6^). Thus, compensatory adaptation was associated with the HOG pathway more commonly than by chance alone.

Previous analyses of multivariate phenotypic divergence among these *A. nidulans* lines suggested that fitness gains were achieved through distinct phenotypic routes (Schoustra *et al.* 2012), but the genetic causes were unknown. The lack of strong parallel evolution provided by our sequence data could indicate that the number of potential genes that allow for compensation is large, or it could be an artifact of our low sample size and lack of complete functional information. Future characterization of mutated genes with unknown function will determine if they interact with the HOG pathway, or if they are generally adaptive and completely independent from the pathway involved with fungicide resistance.

### Comparison of step number for fitness-based and sequence-based estimates

A general result to emerge from genetic studies of adaptation in microbial experiments has been that just a few mutations are usually responsible for the bulk of adaptation achieved during an adaptive walk (Kassen 2014). Indeed, by analyzing fitness data with a maximum likelihood-based approach, we previously estimated that these evolved lines accumulated an average of 2.13 (SE = 0.30) fitness-increasing steps during their adaptive walks (Schoustra *et al.* 2009). The results from our genomic analyses reveal, at first glance, a strikingly different picture. An average of 18.0 (SE = 2.48) mutations were detected in the genome of each evolved line, or 8.45 times greater than the number of steps inferred from fitness trajectory data. The discrepancy between the number of fitness-based adaptive steps and the total number of sequence-based genomic mutations is likely due to two, nonexclusive reasons.

First, not all mutations observed in the evolved lines are adaptive. Some nonadaptive mutations (neutral, nearly neutral, or even slightly deleterious) may become fixed in the population by chance alone, or may hitch-hike to fixation alongside a beneficial mutation. Hitch-hiking is expected to be especially prevalent because reproduction in this experiment was strictly asexual, leading to complete linkage between beneficial mutations and any others that arose. This effect can be quite pronounced: in experimental evolution studies with yeast, for example, typically less than half of the derived genomic mutations have observed adaptive effects (Anderson *et al.* 2010; Kvitek and Sherlock 2011).

Second, not all adaptive mutations are detectable in fitness assays. Large fitness changes will be identified, but adaptive mutations that confer small fitness increases may be below the detection threshold of the phenotypic assays. Also, if multiple beneficial mutations arise in the same genome and fix together (Park and Krug 2007; Jerison and Desai 2015), these would be detected as a single fitness-increasing event. Moreover, fitness measures of mycelial growth rate do not assess all components of total fitness. Mutations that have adaptive effects on other fitness components (*e.g.*, mycelial density, sporulation) would not be detected. In these cases, fitness-based step numbers would underestimated.

Taken together, these explanations suggest that we should be more conservative in our estimate of the number of mutations substituted by focusing attention only on those mutations that are likely to be adaptive. Experimental determination of the individual fitness effects of all 144 derived *A. nidulans* mutations, alone and in combination, is a prodigious task and beyond the scope of the present paper, so we inferred potential adaptive effects based on the mutation location and context within genes. When restricted to mutations occurring within protein-coding DNA, evolved lines had an average of 10.38 (SE = 1.27) mutations, corresponding to 4.87 times the estimated step number. Similarly, when restricted to only those mutations that change the protein sequence, evolved lines had an average of 6.88 (SE = 1.04), or 3.23 times the estimated step number. When restricted to radical amino acid changes (mean = 6.00, SE = 0.96), the difference was only 2.82 times.

**Figure 3:**
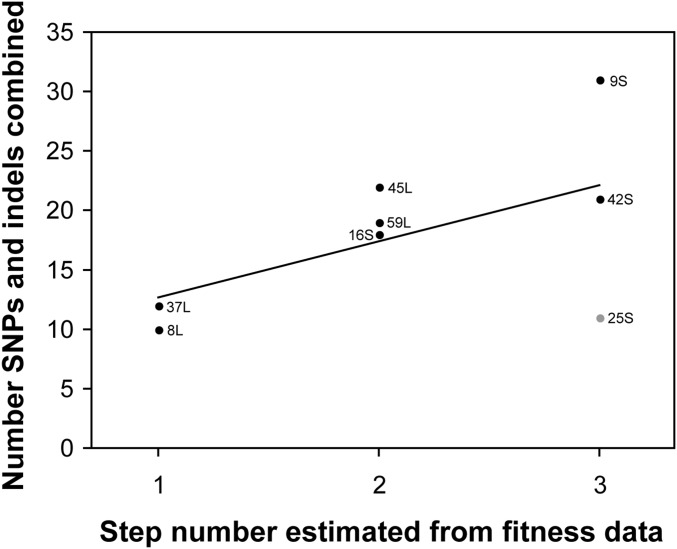
Positive relationship between the estimated step number and number of genomic mutations (SNPs and indels combined) for eight evolved strains (*R* = 0.56, *P* > 0.14). Relationship is significant when outlier strain 25S (shown in gray) is removed (*R* = 0.88, *P* < 0.009). Indel, insertion/deletion; SNP, single nucleotide polymorphism.

Reassuringly, there was a positive correlation between inferred step number and the various measures of genomic mutation number for the evolved lines (Figure 3 and Table 3). Given the low statistical power afforded by a sample size of only eight, it was not surprising that these positive relationships were all statistically nonsignificant. Deviation from linearity was mainly caused by the outlier strain 25S, which had a high predicted number of steps but low number of genomic mutations. Examination of the data revealed that strain 25S had no detected nonsynonymous mutations, but was the only strain that had a predicted structural variant: an inversion that encompassed 13 genes, most of which are in a secondary metabolite gene cluster (AN7884 cluster; Andersen *et al.* 2013). This inversion could be contributing to fitness increases, but is not counted toward genomic mutation numbers. When outlier strain 25S was removed from the analyses, most mutation number measures were significantly correlated with step number (Table 3).

**Table 3:** Correlations between number of estimated steps and other variables

Comparison	Eight Evolved Lines		Seven Evolved Lines*^a^*	
	*R*	*P*	*R*	*P*
Step number *vs.* mycelial growth rate	0.460	0.251	0.330	0.4701
Step number *vs.* number of SNPs	0.534	0.173	0.930	**0.0024**
Step number *vs.* number of SNPs and indel combined	0.562	0.147	0.884	**0.0083**
Step number *vs.* number of SNPs and indels in protein-coding DNA	0.555	0.153	0.970	**0.0003**
Step number *vs.* number of protein-changing SNPs and indels	0.181	0.667	0.689	0.0869
Step number *vs.* number of radical amino acid changes	0.251	0.548	0.689	0.0869

Values in bold indicate *P* < 0.05. SNP, single nucleotide polymorphism.

a Testing for the effect of removing outlier (25S).

### Effect of small *vs.* large population size

The original selection experiment investigated the effects of population size on the properties of adaptive walks. Small (S) and large (L) effective population size treatments were imposed by bottlenecks of either 500 or 50,000 individuals, respectively, at every transfer. The effect of genetic drift was exaggerated in S populations, leading to lower mean fitness and a more rapid increase in the variance of fitness during the experiment (Schoustra *et al.* 2009). What effect does a 100-fold difference in population size have on the spectrum of genomic changes? While we have relatively little statistical power with which to detect effects, we do find some intriguing trends.

First, the variance in total mutation number for the S group is over twice as large as that for the L group (68.9 and 32.2, respectively), although the difference is not statistically significant (*F*-test, *P* > 0.27). Similar patterns are found when each mutation class is analyzed separately. Second, the correlation between fitness-based and sequence-based step number estimates is significantly positive for the L group (*R* = 0.966, *P* < 0.034) but not for the S group (*R* = 0.181, *P* > 0.81), consistent with more pronounced effects of drift at smaller population sizes. Third, we might expect that, after controlling for differences in total mutation number per line, the S lines should have accrued more putatively neutral mutations than the *L* lines. To test this prediction, we calculated the proportion of coding SNPs that were synonymous (silent) and found that S lines did have a greater mean than L lines [0.497 (SE = 0.191) and 0.323 (SE = 0.056), respectively]. Similar results were obtained when we compared the proportion of all mutations that were putatively neutral [intergenic + intron + synonymous; *S* lines = 0.644 (SE = 0.077), L lines = 0.592 (SE = 0.032)]. While these trends are in the predicted direction (*S* > *L*), the differences were not significant (one-tailed *t*-test; *P* > 0.21 and *P* > 0.28, respectively), likely due to the low statistical power afforded by only four lines per treatment.

### Conclusions

Here, we examined the genomic basis of compensatory adaptation to the fitness cost of fungicide resistance in experimentally evolved strains of *A. nidulans*. Our work reveals three important insights into the genetics of adaptive evolution in this system.

First, fludioxonil resistance in the ancestor was likely conferred by a mutation in the histidine kinase *nikA*, the sensor part of the two-component signal transduction phosphorelay system of the HOG MAPK stress response pathway.

Second, to compensate for the pleiotropic negative effects of the resistance mutation, the subsequent fitness gains observed in the evolved lines were likely caused by secondary modification of the HOG MAPK pathway activity. Candidate genes for the compensatory fitness increases were significantly overrepresented by stress response functions, and some mutations were within genes specifically associated with the HOG MAPK pathway itself. We have generated a candidate list of adaptive mutations, however, further detailed genetic and phenotypic analyses are required to confirm the function and fitness effects of each mutation.

Third, there was a positive relationship between the predicted number of adaptive steps, estimated from fitness data, and the number of genomic mutations, determined by whole-genome sequencing. It is clear, however, that fitness-based methods underestimate the number of genomic mutations because they miss mutations of small effect and cannot distinguish fixation events involving multiple mutations. On the other hand, sequencing-based methods likely overestimate the number of adaptive mutations, especially when recombination is absent, because many nonadaptive mutations rise to high frequency by hitchhiking alongside beneficial mutations.

Interestingly, the proportion of mutations predicted to be adaptive in our *Aspergillus* experiment is substantially lower than comparable experiments (Elena and Lenski 2003; Dettman *et al.* 2012; Kassen 2014; Long *et al.* 2015). If we assume, very approximately, that the fitness-based estimates of step number have correctly identified all adaptive mutations, then our sequencing results suggest that, on average, only 12% of the genomic mutations are adaptive. When considering only those mutations that alter the protein sequence, 31% of mutations are adaptive. Such low proportions of adaptive mutations may be due to the higher fraction of the *Aspergillus* genome that is noncoding, compared to bacterial genomes. Also, the spatially structured, multicellular growth form of filamentous fungi may reduce the efficiency of selection. For example, growing as an interconnected mycelial network may hamper competition among contending mutations, and competitive success may depend upon where within the mycelium the new variant arises. These reasons may explain why the proportion of total mutations we predict to be adaptive in *Aspergillus* are low compared to those reported for other, typically unicellular, organisms. In most bacterial experiments, for example, the vast majority of mutations tend to be adaptive (Dettman *et al.* 2012). The generality of our results cannot be determined because, to date, only a few studies have applied the evolve-and-resequence approach to multicellular eukaryotes (*e.g.*, *Drosophila*, Burke *et al.* 2010; Kang *et al.* 2016).

Our study with *Aspergillus* lays out a framework for future research on the genomics of compensatory adaptation. Now that we have identified some clear trends (*e.g.*, stress response and HOG pathway) based on analyses of only eight evolved lines, it will be interesting to see how these patterns hold when the sample size is expanded to dozens or even hundreds of evolved lines. Such increased sample sizes will mitigate our problems with lack of statistical power, and will also allow us to more thoroughly assess the degree of parallelism in genomic evolution during compensatory adaptation.

## Supplementary Material

Supplemental material is available online at www.g3journal.org/lookup/suppl/doi:10.1534/g3.116.036152/-/DC1.
