# Supplementary material for: Genomics of Compensatory Adaptation in Experimental Populations of Aspergillus nidulans
Source: G3 (Bethesda). 2016 Nov 29;7(2):427–36. doi: 10.1534/g3.116.036152 (PMC5295591; doi:10.1534/g3.116.036152)
Supplement: Supplementary file 1 [file 427TableS1.pdf]

**TABLE S1: Strain information for A) sequence data and alignment statistics and B) mycelial growth rate.**

**A) Sequence data and alignment statistics**

| Strain name | Number of reads after trimming | Mean read length after trimming | Proportion reads aligned | Fold mapping coverage |
|---|---|---|---|---|
| WG615 | 14415500 | 99.33 | 0.957 | 46.7 |
| 9S | 43299488 | 74.16 | 0.939 | 102.9 |
| 16S | 31318880 | 74.23 | 0.933 | 74.0 |
| 25S | 35595276 | 74.22 | 0.936 | 84.4 |
| 42S | 38748758 | 74.23 | 0.931 | 91.4 |
| 8L | 34287078 | 74.08 | 0.928 | 86.7 |
| 37L | 38797344 | 74.16 | 0.937 | 92.0 |
| 45L | 43962984 | 74.17 | 0.942 | 104.8 |
| 59L | 27828472 | 74.13 | 0.928 | 65.3 |
| Mean | 34250420 | | 0.937 | 83.1 |

**B) Mycelial growth rate [see Schoustra et al. 2009]**

| Strain name | Colony diameter after 5 days |
|---|---|
| 9S | 60.83 |
| 16S | 38.17 |
| 25S | 64.67 |
| 42S | 43.67 |
| 8L | 33.83 |
| 37L | 51.83 |
| 45L | 47.33 |
| 59L | 66.00 |
