# Supplementary material for: Genomics of Compensatory Adaptation in Experimental Populations of Aspergillus nidulans
Source: G3 (Bethesda). 2016 Nov 29;7(2):427–36. doi: 10.1534/g3.116.036152 (PMC5295591; doi:10.1534/g3.116.036152)
Supplement: Supplementary file 2 [file 427TableS2.pdf]

TABLE S2: Regions of missing coverage for ancestral (WG615) and evolved lines, in relation to the FGSC A4 reference genome.

Scaffold	Missing coverage start	Missing coverage stop	Length
NT_106999	2721	4871	2151
NT_107000	1147	4456	3309
	4867	9072	4205
	9389	21891	12502
	22206	23152	945
	23982	25607	1625
	462147	463854	1708
NT_107001	351111	353374	2264
NT_107002	150222	160740	10519
	274584	280108	5524
	320921	322403	1482
NT_107003	62789	64583	1793
	283297	285854	2557
	342333	345999	3666
	491165	492798	1632
	664761	666555	1794
	759004	761190	2186
NT_107004	10017	42519	32502
	43206	45008	1803
	50817	52706	1889
	87229	92514	5285
	274741	276575	1834
	441518	442179	661
	733374	734119	745
	763330	773457	10127
	942419	945793	3374
	946120	947659	1540
NT_107005	77979	79487	1508
	79964	80898	934
	379962	381723	1761
	445033	449246	4213
	488868	489537	669
	731276	732598	1322
	836093	837956	1863
	864739	866019	1280
	1047000	1047995	995
	1069290	1070174	884
	1113087	1115885	2798
	1144902	1145552	650

NT_107006	308979	309900	921
	1051787	1055180	3393
	1306788	1309196	2408
	1363037	1368276	5239
NT_107007	274246	277660	3413
	382742	384577	1835
	399294	401189	1895
	401433	403162	1729
	521057	522525	1468
	523186	525398	2212
	536419	539559	3140
	542754	545624	2870
	725339	727958	2619
	728205	729741	1537
	754453	755352	900
	883716	885189	1473
	939570	942224	2654
	973561	974184	623
NT_107008	191046	192745	1699
	530964	533730	2766
	940760	945997	5237
	1264784	1265764	980
	1291612	1294758	3146
	1422011	1425528	3518
NT_107009	233074	234776	1702
	525736	526914	1177
	548944	551452	2508
	682213	685974	3761
	751870	755106	3237
	1051248	1052244	996
	1329344	1331999	2655
	1399558	1400060	502
	1844717	1848067	3350
NT_107010	466699	471935	5236
	473149	476493	3344
	476826	479902	3076
	486640	490216	3576
	491146	492380	1235
	492668	493297	629
	494169	500009	5840
	500739	508537	7798
	508792	512546	3754

	512876	513784	908
	517451	518119	668
	552352	553515	1163
	763326	764740	1414
	892049	895077	3028
	1176653	1181894	5240
	1412824	1421943	9119
	1520068	1522538	2471
	1620247	1623078	2831
	1647768	1651138	3370
	1786609	1787139	530
NT_107011	926	2254	1327
	3071	3795	724
	8595	9422	827
	17226	26995	9769
	28181	29631	1450
	40046	42299	2253
	254403	256300	1898
	322212	323956	1744
	540922	547487	6565
	1108432	1112165	3733
	1445750	1447439	1688
	1514124	1515795	1671
	1603640	1609553	5913
	1779564	1781361	1797
	2331031	2331668	637
NT_107012	893	1407	515
	21453	23179	1727
	95239	100480	5241
	177421	179022	1601
	247072	248096	1024
	1053145	1057454	4309
	1112684	1117479	4795
	1318652	1319317	664
	1360958	1364150	3192
	2518761	2520208	1447
	2560704	2561483	779
	2568647	2569337	690
NT_107013	62323	62970	647
	171574	176938	5364
	198698	199337	639
	201287	210053	8766

	337821	338782	962
	341652	342157	505
	345295	350524	5229
	375765	376309	545
	416916	418707	1791
	438555	439413	859
	611932	617300	5368
	644319	647431	3112
	693544	697372	3828
	1386316	1388116	1800
	1388481	1389262	781
	1389543	1390216	673
	1893361	1896021	2659
	2127905	2131159	3254
	2621740	2622717	977
	2664547	2670498	5951
	2694275	2697030	2755
NT_107014	343387	345360	1973
	387692	390133	2441
	618046	624279	6233
	1013530	1014031	502
	1225903	1228395	2492
	1752943	1753917	975
	1845817	1847760	1943
	1927112	1932421	5309
	2462946	2464244	1298
	2719413	2720488	1075
	2766422	2768139	1717
	3193577	3196297	2721
	3411131	3411734	603
NT_107015	4192	5683	1490
	6455	7064	608
	7388	20561	13173
	307825	308340	516
	815972	817752	1780
	913790	919003	5213
	1150199	1150772	573
	1154848	1155520	672
	1179959	1184454	4495
	1959065	1959636	571
	2005968	2011583	5614
	2087093	2088891	1797

	2120382	2122112	1730
	2197848	2199207	1359
	2259633	2264696	5062
	2436101	2441473	5372
	2518453	2519346	892
	2563575	2565367	1791
	2686802	2690039	3238
	3002166	3003965	1799
	3051087	3054623	3536
	3230481	3232047	1567
	3681695	3684214	2519
	4206849	4210817	3968
TOTAL			504347
