# Supplementary material for: Genomics of Compensatory Adaptation in Experimental Populations of Aspergillus nidulans
Source: G3 (Bethesda). 2016 Nov 29;7(2):427–36. doi: 10.1534/g3.116.036152 (PMC5295591; doi:10.1534/g3.116.036152)
Supplement: Supplementary file 3 [file 427TableS3.pdf]

TABLE S3: Regions of poor quality alignment for ancestral (WG615) and evolved lines, in relation to the FGSC A4 reference genome

Scaffold	Approximate start	Approximate stop	Approximate length
NT_106999	none		
NT_107000	316400	316600	200
	556400	558500	2100
NT_107001	0	3400	3400
	479200	481800	2600
NT_107002	800	2700	1900
	3100	3400	300
	3900	5300	1400
NT_107003	205000	209000	4000
	434300	435200	900
	917300	917800	500
NT_107004	none		
NT_107005	1390500	1391900	1400
NT_107006	0	5000	5000
NT_107007	0	2500	2500
	539600	542900	3300
NT_107008	0	2000	2000
	596990	599290	2300
	1480000	1482000	2000
NT_107009	0	2900	2900
	527800	529600	1800
NT_107010	900	2900	2000
	12000	19100	7100
	481580	485180	3600
	514580	515880	1300
	1176900	1181700	4800
	1872300	1872800	500
	2211700	2214300	2600
NT_107011	3400	4900	1500
	40000	42100	2100
NT_107012	0	6200	6200
NT_107013	2773600	2774400	800
	2776200	2776700	500
NT_107014	3051800	3052900	1100
	3796600	3798000	1400
NT_107015	0	1800	1800
	1665510	1667610	2100
	4303500	4306200	2700
