# Supplementary material for: Genomics of Compensatory Adaptation in Experimental Populations of Aspergillus nidulans
Source: G3 (Bethesda). 2016 Nov 29;7(2):427–36. doi: 10.1534/g3.116.036152 (PMC5295591; doi:10.1534/g3.116.036152)
Supplement: Supplementary file 4 [file 427TableS4.pdf]

TABLE S4: Protein-changing differences between FGSC A4 and WG615 within the genomic region to which fludioxonil resistance was mapped.

Position in NT_107011	FGSC A4 state	WG615 state	Effect on gene	Gene	Gene product
89892	*	-TGA	CODON_DELETION	AN4319	uncharacterized
92319	*	+T	FRAME_SHIFT	AN4320	uncharacterized, predicted role in chromatin remodeling and nuclear chromosome localization
212031	A	G	NON_SYNONYMOUS_CODING	AN4359	uncharacterized, predicted zinc ion binding activity
577407	G	C	NON_SYNONYMOUS_CODING	AN4479	**nikA, putative histidine-specific protein kinase, part of a two-component signal transduction phosphorelay system involved in response to fungicides; mutant has a strong growth defect**
612858	G	T	NON_SYNONYMOUS_CODING	AN4489	uncharacterized, predicted DNA binding, RNA polymerase II transcription factor activity
840816	G	A	NON_SYNONYMOUS_CODING	AN4564	teaA, cell-end marker protein
847809	C	G	NON_SYNONYMOUS_CODING	AN4566	chsC, class I chitin synthase with a role in chitin biosynthesis
896495	*	-CT	FRAME_SHIFT	AN4588	uncharacterized
899726	*	-C	FRAME_SHIFT	AN4590	uncharacterized, putative sugar transporter
1064579	G	T	NON_SYNONYMOUS_CODING	AN4643	CYP675A1, putative cytochrome P450
1305482	G	T	NON_SYNONYMOUS_CODING	AN4721	uncharacterized, putative U2-type spliceosomal complex localization
1306254	C	G	NON_SYNONYMOUS_CODING	AN4721	uncharacterized, putative U2-type spliceosomal complex localization
1308961	*	-T	FRAME_SHIFT	AN4722	uncharacterized, predicted glycosylphosphatidylinositol (GPI)-anchored protein
