# Supplementary material for: Genomics of Compensatory Adaptation in Experimental Populations of Aspergillus nidulans
Source: G3 (Bethesda). 2016 Nov 29;7(2):427–36. doi: 10.1534/g3.116.036152 (PMC5295591; doi:10.1534/g3.116.036152)
Supplement: Supplementary file 5 [file 427TableS5.pdf]

TABLE S5: Information on the derived mutations that affect genes. Gene product and function were determined from automated annotation, homology searches, and literature searches.

Strain	Scaffold	Position	Ancestral state	Derived state	Effect on gene	Change	Gene	Gene product	Gene function
9S	NT_107004	901609	A	G	NON_SYNONYMOUS_CODING	L/S	AN8562	ankyrin repeat protein	transcript upregulated in response to camptothecin
9S	NT_107005	423256	C	T	SYNONYMOUS_CODING	I/I	AN7991	uncharacterized	
9S	NT_107009	624073	G	A	SYNONYMOUS_CODING	A/A	AN5987	uncharacterized	
9S	NT_107011	1125770	A	G	SYNONYMOUS_CODING	F/F	AN4668	mpkC, mitogen activated protein kinase (MAPK), highly similar to HogA	response to osmotic stress, development
9S	NT_107011	1813800	G	T	NON_SYNONYMOUS_CODING	R/L	AN4886	putative Golgi complex component Cog3	intracellular protein transport, macroautophagy, peroxisome degradation
9S	NT_107011	2468074	G	A	NON_SYNONYMOUS_CODING	R/K	AN5091	llmE, putative LaeA-like methyltransferase	negative regulator of sterigmatocystin production and sexual development
9S	NT_107012	1582116	C	T	NON_SYNONYMOUS_CODING	P/L	AN3967	uncharacterized	
9S	NT_107013	294756	G	A	NON_SYNONYMOUS_CODING	G/S	AN2699	uncharacterized	predicted transmembrane transport activity, integral component of membrane localization
9S	NT_107013	360075	C	T	SYNONYMOUS_CODING	V/V	AN2717	rrpC, putative RNA-directed RNA polymerase	RNA-directed RNA polymerase activity
9S	NT_107013	784131	GCGAATGCG-AGTTAGATAC-CAAGCCG-AGACG	GCG	FRAME_SHIFT	-28	AN2847	putative recombination hotspot-binding protein (translin)	predcited RNA binding, single-stranded telomeric DNA binding activity, role in tRNA processing
9S	NT_107013	2048460	CCAAGCCTG	C	FRAME_SHIFT	-8	AN3247	ABC multidrug transporter	predicted ATP binding, ATPase activity, coupled to transmembrane movement of substances
9S	NT_107013	2050078	C	T	SYNONYMOUS_CODING	F/F	AN3247	ABC multidrug transporter	predicted ATP binding, ATPase activity, coupled to transmembrane movement of substances
9S	NT_107013	2059735	C	T	NON_SYNONYMOUS_CODING	G/S	AN3251	uncharacterized	
9S	NT_107014	1146707	C	T	NON_SYNONYMOUS_CODING	P/S	AN1772	faeB, feruloyl esterase type B	degradation of xylan
9S	NT_107014	2100812	G	C	NON_SYNONYMOUS_CODING	E/D	AN2078	uncharacterized	predicted RNA polymerase II transcription cofactor activity, role in regulation of transcription from RNA polymerase II promoter and mediator complex localization
9S	NT_107015	129973	C	G	INTRON		AN0035	uncharacterized	
9S	NT_107015	3316002	T	A	NON_SYNONYMOUS_CODING	L/H	AN1071	uncharacterized	Predicted ADP binding, catalytic activity, microtubule motor activity, nucleoside-triphosphatase activity, nucleotide binding activity, role in nucleoside metabolic process and kinesin complex localization
16S	NT_107003	299918	G	A	NON_SYNONYMOUS_CODING	L/F	AN8671	uncharacterized	protein domain specific binding activity
16S	NT_107007	387388	G	T	NON_SYNONYMOUS_CODING	H/N	AN7091	uncharacterized	predicted ATP binding, nucleoside-triphosphatase activity
16S	NT_107009	2127484	C	T	NON_SYNONYMOUS_CODING	S/L	AN11924	uncharacterized	predicted oxidoreductase activity, iron ion binding
16S	NT_107010	1900601	C	T	NON_SYNONYMOUS_CODING	P/S	AN5687	uncharacterized	
16S	NT_107011	2057577	G	A	NON_SYNONYMOUS_CODING	R/C	AN10620	uncharacterized	
16S	NT_107012	109459	G	A	NON_SYNONYMOUS_CODING	A/T	AN3504	putative alpha-1,4-glucosidase	starch and disaccharide degradation
16S	NT_107012	1024725	C	T	NON_SYNONYMOUS_CODING	S/F	AN3793	ppzA, serine/threonine-protein phosphatase, catalytic subunit of protein phosphatase Z (PPZ)	resistance to oxidative stress
16S	NT_107013	664345	G	T	SYNONYMOUS_CODING	P/P	AN10343	uncharacterized	putative phosphate transport activity
16S	NT_107013	914927	G	A	NON_SYNONYMOUS_CODING	S/F	AN2891	putative LaeA-like methyltransferase	putative methyltransferase activity
16S	NT_107013	2227908	G	A	NON_SYNONYMOUS_CODING	T/I	AN3307	agsB, catalytic subunit of the major alpha-1,3 glucan synthase complex	glucan synthesis, osmotic stress and cell wall integrity
16S	NT_107014	47505	G	T	INTRON		AN1420	sip5, C2H2 zinc finger protein	cellular response to glucose starvation
16S	NT_107015	21195	G	C	NON_SYNONYMOUS_CODING	A/P	AN0005	lbuA, Lactobacillus up-regulated protein	
25S	NT_107010	262330	G	A	SYNONYMOUS_CODING	V/V	AN5174	atg5, autophagy protein	autophagy, ligase activity, C-terminal protein lipidation, cytoplasm to vacuole transport
25S	NT_107010	862757	C	T	SYNONYMOUS_CODING	L/L	AN5370	putative MFS multidrug transporter	predicted transmembrane transport
25S	NT_107011	65892	G	A	SYNONYMOUS_CODING	F/F	AN4312	uncharacterized	
25S	NT_107011	428165	ACCCCCCCC	ACCCCCCCCC	FRAME_SHIFT	+1	AN4431	uncharacterized	predicted RNA binding activity

25S	NT_107011	2400837	ACCC	ACC	FRAME_SHIFT	-1	AN5072	uncharacterized	
25S	NT_107014	1397849	T	C	SYNONYMOUS_CODING	S/S	AN1862	uncharacterized	
42S	NT_107003	56712	G	A	SYNONYMOUS_CODING	I/I	AN8596	putative zinc-binding transcription factor	predicted sequence-specific DNA binding activity
42S	NT_107004	79979	C	T	NON_SYNONYMOUS_CODING	G/S	AN8304	uncharacterized	predicted zinc ion binding activity
42S	NT_107004	130125	C	T	SYNONYMOUS_CODING	G/G	AN8314	uncharacterized	predicted hydrolase activity
42S	NT_107008	799840	G	T	SYNONYMOUS_CODING	T/T	AN6753	putative NADH-dependent flavin oxidoreductase	stress response (menadione)
42S	NT_107009	1011536	C	A	NON_SYNONYMOUS_CODING	H/N	AN6120	putative ADP ribosylation factor guanine nucleotide exchange factor (Arf GEF)	vesicular trafficking
42S	NT_107009	1072972	C	T	SYNONYMOUS_CODING	G/G	AN6134	uncharacterized	
42S	NT_107010	232260	A	C	NON_SYNONYMOUS_CODING	E/A	AN5167	uncharacterized	predicted phospholipid binding, zinc ion binding activity
42S	NT_107010	1715175	C	T	INTRON		AN5627	sonB, nuclear pore complex protein	
42S	NT_107013	975782	A	C	NON_SYNONYMOUS_CODING	Q/P	AN2911	atfA, basic-region leucine zipper transcription factor activated by HOG MAPK	stress response (osmotic, fungicide, oxidative)
42S	NT_107013	2028451	G	A	INTRON		AN3240	uncharacterized	
42S	NT_107013	2474957	G	T	STOP_GAINED	G/*	AN3374	uncharacterized	
42S	NT_107014	966767	G	A	SYNONYMOUS_CODING	L/L	AN1700	putative 26S proteasome regulatory subunit Rpn2	proteasome assembly, stress response, transcript upregulated in response to camptothecin
42S	NT_107015	902272	T	A	NON_SYNONYMOUS_CODING	V/E	AN0286	uncharacterized	
42S	NT_107015	1221587	G	T	NON_SYNONYMOUS_CODING	K/N	AN0391	putative NACHT domain protein	
42S	NT_107015	2275520	C	T	SYNONYMOUS_CODING	I/I	AN10118	uncharacterized	predicted mannosyltransferase, endoplasmic reticulum localization
8L	NT_107004	620111	C	T	SYNONYMOUS_CODING	A/A	AN8470	uncharacterized	predicted oxidoreductase activity and role in metabolic process
8L	NT_107009	178305	C	A	NON_SYNONYMOUS_CODING	N/K	AN5849	uncharacterized	predicted sequence-specific DNA binding RNA polymerase II transcription factor activity, zinc ion binding activity
8L	NT_107011	1173060	C	T	SYNONYMOUS_CODING	L/L	AN4686	csnA, chitosanase	endo-chitosanase activity, predicted glycosyl phosphatidylinositol (GPI)-anchor
8L	NT_107012	83807	C	T	SYNONYMOUS_CODING	N/N	AN3496	inpB, putative nonribosomal peptide synthetase, secondary metabolite gene cluster member	predicted role in asperfuranone biosynthesis
8L	NT_107012	2309740	G	A	NON_SYNONYMOUS_CODING	G/D	AN4206	DnaJ domain protein	protein folding and chaperone binding, stress response, transcript upregulated in response to camptothecin
8L	NT_107014	1128177	G	A	NON_SYNONYMOUS_CODING	E/K	AN1764	uncharacterized	
8L	NT_107014	2100812	G	C	NON_SYNONYMOUS_CODING	E/D	AN2078	uncharacterized	predicted RNA polymerase II transcription cofactor activity, role in regulation of transcription from RNA polymerase II promoter and mediator complex localization
37L	NT_107005	129881	G	A	SYNONYMOUS_CODING	K/K	AN7884	putative nonribosomal peptide synthase	siderophore biosynthesis
37L	NT_107007	1350709	G	A	NON_SYNONYMOUS_CODING	G/E	AN7396	bglM, putative beta-glucosidase	cellulose and polysaccharide degradation
37L	NT_107009	804456	C	G	INTRON		AN6052	uncharacterized	
37L	NT_107010	391404	GACC	GACCACC	CODON_CHANGE_PLUS_CODON_INSERTION	P/HP	AN5217	pilA, putative eisosome component	protein localization, stress response, transcript upregulated in response to camptothecin
37L	NT_107011	1496118	C	A	NON_SYNONYMOUS_CODING	L/I	AN4789	uvsI, DNA polymerase	response to UV-damage of DNA, post-replication DNA repair
37L	NT_107012	420568	C	T	NON_SYNONYMOUS_CODING	D/N	AN3601	uncharacterized	predicted catalytic activity
37L	NT_107013	1568477	G	A	NON_SYNONYMOUS_CODING	T/I	AN3101	phkB, histidine kinase D5, stress-activated phosphotransfer protein	phosphorelay sensor kinase activity, stress-activated (osmotic, oxidative, fungicide)
37L	NT_107014	2500192	G	A	NON_SYNONYMOUS_CODING	S/L	AN2204	osmoadaptation protein with unknown function, stress response	cellular response to osmotic stress
45L	NT_107005	1149835	G	A	SYNONYMOUS_CODING	I/I	AN8215	metA, putative methylenetetrahydrofolate reductase (NADPH)	predicted role in one-carbon metabolism, induced by homocysteine

45L	NT_107008	73412	A	C	SYNONYMOUS_CODING	P/P	AN10832	putative RNA polymerase II elongator subunit	regulation of transcription from RNA polymerase II promoter
45L	NT_107009	1531307	G	C	NON_SYNONYMOUS_CODING	P/R	AN6280	uncharacterized	stress response, transcript upregulated in response to camptothecin
45L	NT_107011	53560	G	A	NON_SYNONYMOUS_CODING	P/S	AN4308	50S ribosomal protein L3	structural constituent of ribosome activity and mitochondrial large ribosomal subunit
45L	NT_107013	1615005	C	T	SYNONYMOUS_CODING	I/I	AN3113	ugtA, UDP-galactofuranose transporter, DMT family organic anion transporter, multidrug resistance efflux domain	involved in development of conidiophore and conidia, cell wall architecture, hyphal morphology, and drug sensitivity
45L	NT_107013	2478684	C	T	SYNONYMOUS_CODING	L/L	AN10396	putative farnesyl-diphosphate farnesyltransferase	lipid biosynthetic process
45L	NT_107014	2269967	G	A	STOP_GAINED	R/*	AN2130	putative cell division control protein Cdc25	predicted Ras guanyl-nucleotide exchange factor activity
45L	NT_107014	2468260	C	G	NON_SYNONYMOUS_CODING	E/Q	AN2194	uncharacterized	predicted serine-type endopeptidase activity and role in proteolysis
45L	NT_107014	2642511	C	A	NON_SYNONYMOUS_CODING	P/Q	AN2254	uncharacterized	
45L	NT_107014	2751563	A	C	NON_SYNONYMOUS_CODING	V/G	AN2287	putative transmembrane transporter	predicted amino acid transport activity, GABA transport activity
45L	NT_107015	2059816	G	C	NON_SYNONYMOUS_CODING	G/A	AN0660	furA, putative nucleobase and allantoin transporter	induced by allantoin and by uric acid
45L	NT_107015	3761006	C	T	NON_SYNONYMOUS_CODING	S/F	AN1217	putative LIM/homeobox transcription factor	stress response, transcript upregulated in response to camptothecin
59L	NT_107003	70449	G	A	NON_SYNONYMOUS_CODING	S/L	AN11099	uncharacterized	predicted sequence-specific DNA binding RNA polymerase II transcription factor activity, zinc ion binding activity
59L	NT_107003	577656	GTTT	GTT	FRAME_SHIFT	- 1	AN8765	activator of chitin synthase	response to osmotic stress, regulation of chitin synthase activity and cell wall formation
59L	NT_107006	811136	C	T	NON_SYNONYMOUS_CODING	S/L	AN7662	freA, putative metalloreductase	predicted role in cellular iron ion homeostasis, hyphal tip growth
59L	NT_107008	789270	G	A	NON_SYNONYMOUS_CODING	H/Y	AN6749	llmF, putative LaeA-like methyltransferase	negative regulator of sterigmatocystin production and sexual development
59L	NT_107010	661252	C	T	NON_SYNONYMOUS_CODING	S/L	AN5300	putative aconitate hydratase	predicted role in the TCA cycle
59L	NT_107011	733380	G	A	SYNONYMOUS_CODING	I/I	AN4526	pttA, putative thiamine transporter	nucleoside transport
59L	NT_107014	2368227	C	T	SYNONYMOUS_CODING	I/I	AN2158	putative threonine dehydrogenase	predicted role in glycine, serine, and threonine metabolism
59L	NT_107014	3422781	G	A	NON_SYNONYMOUS_CODING	H/Y	AN2492	uncharacterized	predicted function in fungal-type cell wall organization or biogenesis
59L	NT_107014	3647357	C	T	SYNONYMOUS_CODING	P/P	AN2555	serine-type carboxypeptidase	regulation of cellular fusion
59L	NT_107015	4019225	C	A	NON_SYNONYMOUS_CODING	G/V	AN10172	uncharacterized	predicted dicarboxylic acid transmembrane transporter activity, role in mitochondrial transport and mitochondrial inner membrane localization
