# Supplementary material for: Genomics of Compensatory Adaptation in Experimental Populations of Aspergillus nidulans
Source: G3 (Bethesda). 2016 Nov 29;7(2):427–36. doi: 10.1534/g3.116.036152 (PMC5295591; doi:10.1534/g3.116.036152)
Supplement: Supplementary file 6 [file 427TableS6.pdf]

TABLE S6: Information on A) derived mutations in intergenic regions and B) predicted structural variants

A) Derived mutations in intergenic regions

Strain	Scaffold	Position	Ancestral state	Derived state	In 5'-UTR	In potential promoter region*	Downstream gene	Gene function
**9S**	NT_107000	90973	G	A				
**9S**	NT_107005	352109	C	G				
**9S**	NT_107006	1132739	T	A				
**9S**	NT_107009	1586399	T	C				
**9S**	NT_107010	1686757	G	A				
**9S**	NT_107012	623930	T	C		YES	AN3666	uncharacterized, putative role in DNA strand elongation involved in mitotic DNA replication, PSF1 domain protein
**9S**	NT_107013	352454	G	T				
**9S**	NT_107013	352455	G	A				
**9S**	NT_107013	352457	A	G				
**9S**	NT_107014	645215	C	T				
**9S**	NT_107014	1479678	C	A		YES	AN1892	mitochondrial import inner membrane translocase subunit TIM17
**9S**	NT_107014	1480202	A	T				
**9S**	NT_107014	3579237	G	A				
**9S**	NT_107015	1304920	GCCCCCCC	GCCCCCC				
**16S**	NT_107011	414917	G	T	YES		AN4426	uncharacterized, tyrosine phosphatase family protein
**16S**	NT_107012	864890	G	A				
**16S**	NT_107012	2061036	A	G		YES	AN4129	uncharacterized, putative transporter
**16S**	NT_107013	455424	G	A				
**16S**	NT_107013	1284785	G	A		YES	AN3009	uncharacterized
**16S**	NT_107015	24863	T	C				
**25S**	NT_107011	1269150	G	A		YES	AN4707	uncharacterized, putative pre-mRNA-splicing factor.
**25S**	NT_107012	78877	G	T		YES	AN3495	inpA, putative nonribosomal peptide synthase
**25S**	NT_107014	325727	G	A				
**25S**	NT_107014	1067021	G	A				
**25S**	NT_107015	347374	C	T				

**42S**	NT_107001	211336	T	C				uncharacterized, enoyl-CoA hydratase/isomerase family protein
**42S**	NT_107009	432466	C	T				
**42S**	NT_107009	478920	C	T				
**42S**	NT_107010	1926102	G	A				
**42S**	NT_107012	196754	G	T				
**42S**	NT_107013	966380	G	C				
**8L**	NT_107005	148698	C	T				uncharacterized
**8L**	NT_107009	796042	G	A				
**8L**	NT_107012	95293	C	T				uncharacterized
**37L**	NT_107003	450076	T	C				
**37L**	NT_107005	1243348	G	A	YES		AN8246	srpA, putative signal recognition particle protein
**37L**	NT_107009	1043581	C	T	YES		AN6127	uncharacterized, putative vacuolar ATP synthase 16 kDa proteolipid subunit
**37L**	NT_107012	1317769	C	T				
**45L**	NT_107005	102001	G	A				uncharacterized, putative fatty-acyl-CoA synthase, subunit alpha
**45L**	NT_107007	165857	A	C				
**45L**	NT_107008	569650	C	T				
**45L**	NT_107009	564142	C	T	YES		AN5967	uncharacterized
**45L**	NT_107011	561576	G	A				
**45L**	NT_107011	1163714	G	C				uncharacterized, putative oligosaccharyltransferase beta subunit
**45L**	NT_107011	1471498	CCGCCGGTCGC	CC		YES	AN4780	uncharacterized, putative pyridoxamine phosphate oxidase
**45L**	NT_107013	1190589	GCTCCGCAATC	GCTCCGCAATCTCCGCAATC		YES	AN2971	uncharacterized
**45L**	NT_107013	2018595	G	A				
**45L**	NT_107014	3332192	C	T				
**59L**	NT_107004	53945	G	A				
**59L**	NT_107007	527780	C	T				
**59L**	NT_107010	1404962	G	A				
**59L**	NT_107012	1622324	G	A				
**59L**	NT_107013	2415505	G	A				
**59L**	NT_107014	891837	G	A				

**59L**	NT_107014	2155614	C	T	YES	AN2095	uncharacterized, putative HIT finger domain protein
**59L**	NT_107015	2876257	G	A			
**59L**	NT_107015	993372	CGGG	CGG			

* less than 100 bp upstream of transcription start site.

B) Predicted, derived structural variants

**Strain**	**Scaffold**	**Type of variant**	**Predicted position**	**Genes disrupted by breakpoint**	**Genes within variant**
**25S**	NT_107005	inversion	39616 – 141689	none	AN7861 – AN7881, AN11028, AN11031, AN7883 – AN7885
