# Supplementary material for: Genomics of Compensatory Adaptation in Experimental Populations of Aspergillus nidulans
Source: G3 (Bethesda). 2016 Nov 29;7(2):427–36. doi: 10.1534/g3.116.036152 (PMC5295591; doi:10.1534/g3.116.036152)
Supplement: Supplementary file 7 [file 427TableS7.pdf]

TABLE S7: Number of mutations and mutation rates for *A. nidulans* chromosomes

Chromosome	Included scaffolds	Number of nucleotides	Number of mutations in evolved lines	Mutations per nucleotide per generation ($X10^{-10}$)
**I**	NT_107008, NT_107009	3682981	17	7.21
**II**	NT_107005, NT_107012	3963651	21	8.28
**III**	NT_107003, NT_107011	3396693	21	9.66
**IV**	NT_107006, NT_107007	2823533	6	3.32
**V**	NT_107004, NT_107010	3173690	15	7.38
**VI**	NT_107001, NT_107013	3348957	25	11.66
**VII**	NT_107002, NT_107014	4442834	24	8.44
**VIII**	NT_107000, NT_107015	4866365	15	4.82
**All**	All	29698704	144	7.58
