# Supplementary material for: Genomics of Compensatory Adaptation in Experimental Populations of Aspergillus nidulans
Source: G3 (Bethesda). 2016 Nov 29;7(2):427–36. doi: 10.1534/g3.116.036152 (PMC5295591; doi:10.1534/g3.116.036152)
Supplement: Supplementary file 8 [file 427TableS8.pdf]

TABLE S8: Summary of functional information for the genes mutated in evolved lines. To focus on main functional categories, only GO Slim categories containing at least 4 genes are shown.

GO Slim set	GO Slim number	GO Slim term	Number of genes
Cellular component	16020	membrane	16
	5634	nucleus	16
	5829	cytosol	9
	12505	endomembrane system	7
	5739	mitochondrion	5
	5886	plasma membrane	5
	5783	endoplasmic reticulum	4
Biological process	50789	regulation of biological process	16
	6810	transport	12
	6950	response to stress	12
	42221	response to chemical	7
	5975	carbohydrate metabolic process	5
	16070	RNA metabolic process	5
	32502	developmental process	5
	6464	cellular protein modification process	5
	7165	signal transduction	4
Molecular function	16787	hydrolase activity	11
	16740	transferase activity	8
	16491	oxidoreductase activity	7
	3677	DNA binding	5
	5215	transporter activity	5
	3723	RNA binding	4
